# Supplementary material for: Extracellular Signalling Modulates Scar/WAVE Complex Activity through Abi Phosphorylation
Source: Cells. 2021 Dec 10;10(12):3485. doi: 10.3390/cells10123485 (PMC8700165; doi:10.3390/cells10123485)
Supplement: Supplementary file 1 [file cells-10-03485-s001.zip › cells-1439571 supplementary/cells-1439571-supplementary.pdf]

cells MDPI

## Supplementary Materials

```
Dictyostelium    6    DINVYSQTTIPNAMAELMDNHNKMEQISAYCKSLYA-NGDAAQAYEQTQGYAKNALLNVA   64
                      ++ +  +  IP     L D++  +E+++ YC++ Y  + D  +A E+T+ Y   +L +VA
H. sapiens       3    ELQMLLEEEIPGGRRALFDSYTNLERVADYCENNYIQSADKQRALEETKAYTTQSLASVA   62

Dictyostelium    65   YHIQTVGTHITSLLQLQTNEMEKLNIEIQTLTQRVRMIHDSTGTNVFSNQDAAKPYKSSL  124
                      Y I T+  ++  +L +Q +++ ++    I  ++Q V +  +              K   +S
H. sapiens       63   YLINTLANNVLQMLDIQASQLRRMESSINHISQTVDIHKEKVARREIGILTTNK--NTSR  120

                                                                     166 168 169
Dictyostelium    125  KNRKVDTEATKAPVKYVHKPISYGISASDINQNGVPPPLNHSNSSANL  172
                       ++ +     + PV+Y+ KPI Y I   DI  +GV    L     S+ N+
H. sapiens       121  THKIIAPANLERPVRYIRKPIDYTI-LDDIG-HGVKWLLRFKVSTQNM  166
```

**Figure S1.** Amino acid sequence comparison of *Dictyostelium* Abi with *Homo sapiens* Abi2. *Dictyostelium* Abi amino acid sequence was BLAST searched against *H. sapiens* which resulted in Abi2 as the nearest homolog with 24% identity and 49% similarity (E value=4-18). Conserved serine and threonine residue is indicated in red brackets.

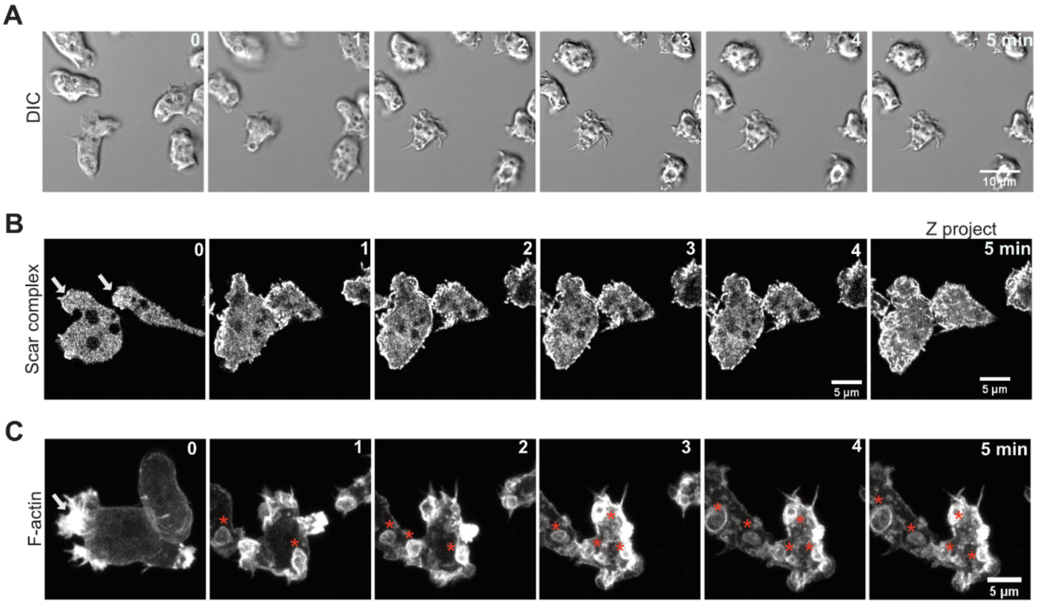


**Figure S2**. Effect of osmolarity on cell shape, Scar/WAVE and Actin localization. (**A**) Retraction of pseudopods and shrinkage of cells after addition of sorbitol (0.4 M). Cells in glass-bottom dishes were imaged by DIC microscopy at a frame interval of 2 s (Scale bar = 10 mm). (**B**) Distribution of the Scar/WAVE complex after sorbitol treatment. Cells expressing EGFP-Nap1, a marker of the Scar/WAVE complex, were imaged by AiryScan confocal microscopy, and 0.4 M sorbitol was added during imaging. Sorbitol inhib localization of the Scar/WAVE complex from pseudopodia and causes relocation to the cell cortex (Scale bar = 5 mm). (**C**) Distribution of actin after sorbitol treatment. Cells expressing lifeact-mRFPmars2 were imaged by AiryScan confocal microscopy. Actin relocates to macropinosomes/vesicles (*) after the addition of sorbitol (Scale bar = 5 mm).

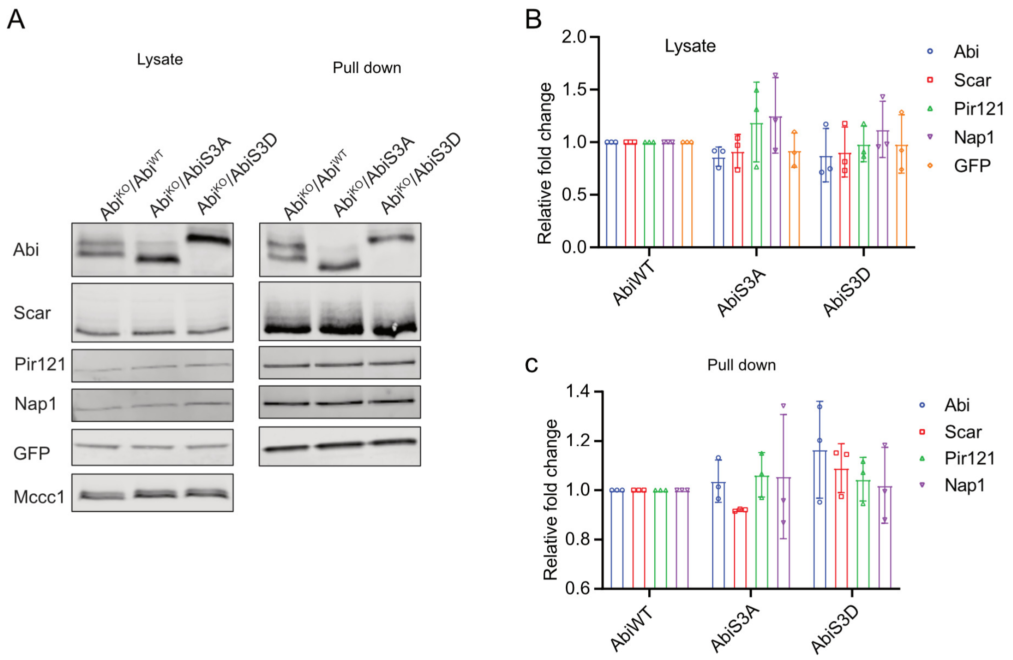


**Figure S3.** Expression pattern and effect of Abi$^{WT}$, Abi$^{S3A}$ and Abi$^{S3D}$ in the Scar/WAVE complex formation. (**A**) Abi$^{WT}$, Abi$^{S3A}$ and Abi$^{S3D}$ were co-expressed with HSPC300-eGFP in Abi- cells, and cell lysates were immunoprecipitated using GFP-TRAP. Lysate and pull-down samples were analyzed for the expression of Abi, Scar, Pir121, Nap1 and GFP by Western blotting. (**B**,**C**) Quantification of Western blots shows that Abi$^{WT}$, Abi$^{S3A}$ and Abi$^{S3D}$ formed stable complexes.

**Video S1:** Recruitment of the Scar/WAVE complex after folate treatment. eGFP-Nap1 cells were seeded on Lab-Tek II coverglass chambers and imaged with an AiryScan microscope. Folate (200 μM) was added to the cells undergoing imaging. Filmed at 1 frame/3 s, the movie shows 10 frames/second. Folate was added after frame 4 (after 12 s).

**Video S2:** Recruitment of the Scar/WAVE complex after cAMP treatment. eGFP-Nap1 cells were seeded on Lab-Tek II coverglass chambers and imaged by AiryScan confocal microscopy. cAMP (10 μM) was added to the cells undergoing imaging. Filmed at 1 frame/3 s, the movie shows 10 frames/second. cAMP was added after frame 4 (after 12 s).

**Video S3:** Change in cell morphology after sorbitol treatment. *Dictyostelium* cells were seeded on Lab-Tek II coverglass chambers and imaged by DIC microscopy (60x). Filmed at 1 frame/1 s, the movie shows 10 frames/second.

**Video S4:** Recruitment of the Scar/WAVE complex after sorbitol treatment. eGFP-Nap1 cells were seeded on Lab-Tek II coverglass chambers and imaged by AiryScan confocal microscopy. Sorbitol (0.4 M) was added to the cells undergoing imaging. Filmed at 1 frame/3 s, the movie shows 10 frames/second.

**VideoS5:** Recruitment of F-actin after sorbitol treatment. Life-act-eGFP expressing cells were seeded on Lab-Tek II coverglass chambers and imaged by AiryScan confocal microscopy. Sorbitol (0.4 M) was added to the cells undergoing imaging. Filmed at 1 frame/3 s, the movie shows 10 frames/second.

**Video S6:** Recruitment of the Scar/WAVE and Arp2/3 complexes after latrunculin treatment. eGFP-Nap1 cells expressing ArpC2-mRFPmars 2 were seeded on Lab-Tek II coverglass chambers and imaged by AiryScan confocal microscopy. LatrunculinA (5 μM) was added to the cells undergoing imaging. Filmed at 1 frame/3 s, the movie shows 5 frames/second. Latrunculin was added after frame 1 (after 1 min).

**Video S7:** Scar complex activation in Abi phosphomutants. $Abi^{KO}$ cells co-expressing HSPC300-eGFP and Abi were allowed to migrate under agarose up folate gradient and Scar complex activation in pseudopods were observed by AiryScan confocal microscopy. Imaged at 1 frame/3 s, the movie shows 10 frames/second.

**Video S8:** Pseudopod formation in Abi phosphomutants. $Abi^{KO}$ cells expressing $Abi^{WT}$, $Abi^{S3A}$ and $Abi^{S3D}$ were allowed to migrate under agarose up a folate gradient and observed by DIC microscopy. Filmed at 1 frame/2 s, the movie shows 10 frames/second.

**Video S9:** Scar complex activation in Abi phosphomutants. Scar-/Abi-/Pir121-eGFP cells expressing $Scar^{WT}$/$Abi^{WT}$, $Scar^{S8A}$/$Abi^{S3A}$ and $Scar^{S8D}$/$Abi^{S3D}$ were allowed to migrate under agarose up folate gradient and Scar complex activation in pseudopods were observed by AiryScan confocal microscopy. Imaged at 1 frame/3 s, the movie shows 10 frames/second.

**Video S10:** Pseudopod formation in Scar and Abi phosphomutants. Scar-/Abi- cells expressing $Scar^{WT}$/$Abi^{WT}$, $Scar^{S8A}$/$Abi^{S3A}$ and $Scar^{S8D}$/$Abi^{S3D}$ were allowed to migrate under agarose up a folate gradient and observed by DIC microscopy. Filmed at 1 frame/2 s, the movie shows 10 frames/second.

**Table S1.** Cell lines used in this study.

| No. | Cell line | Given Name | Reference |
|---|---|---|---|
| 1. | Ax3 | | dictyBase |
| 2. | NC4 | | dictyBase |
| 3. | Abi- | Ap5b | [1] |
| 4. | eGFP-Nap1 | - | [2] |
| 5. | Scar-/Abi- | SP2 | This study |
| 6. | Scar-/Abi-/Pir121-eGFP | SP21 | This study |
| 7. | Pir121- | SP3 | [3] |
| 8. | Pir121-eGFP WT | SP7 | [3] |
| 9. | Pir121-eGFP A site | SP11 | [3] |

**Table S2.** List of primers.

| Amplicon Name | Forward Primer (5′-3′) | Reverse Primer (5′-3′) |
|---|---|---|
| Abi 5′UTR | GAATTCGCCGGCG-GAGGGAAAAATTTTAGAGGA | GCATTTGGAATTGTGGTTT-GTGAATAAACG |
| Abi S166, 168, 169A | GTTCCACCACCATT-GAATCATGCAAATGCAG-CAGCTAATTTAACATCATCAAG | CTTGATGATGTTAAATT-AGCTGCTGCATTTGCATGAT-TCAATGGTGGTGGAAC |
| Abi S166, 168, 169D | GATAATGACGAC-GCTAATTTAACATCATCAAGTG | ATTAGCGTCGTCATTATCATGATTCAATGGTGGTGGAAC |
| Pir121 3′UTR | CAAAAAGATAAA-GCTTATCGCCAC-CACCCCCACCAATGTAA | TACGCAAACCGCCTCTGCCG-GAATTCTTGA-GAACTCCCTATCTTTATGATT |
| Blasticidin (BSR) | GCAGAAGCCATT-GCGATTGGT | ACCAATCG-CAATGGCTTCTGC |
| Abi 5′UTR | CCAATAGTAACAC-TATCTGCCAT | |
| Abi 3′UTR | | TATTAAAGGATGGTGG-TATATTC |

**Table S3.** Plasmids created and used in this study.

| No. | Name of the Plasmid | Gene/s |
|---|---|---|
| 1. | pSP287 | Shuttle vector of Abi WT |
| 2. | pSP318 | Pir121eGFP knock in vector |
| 3. | pSP384 | Abi WT |
| 4. | pSP396 | Abi S166, 168, 169A (S3A) |
| 5. | pSP427 | Abi S166, 168, 169D (S3D) |
| 6. | pSP428 | Abi WT and HSPC300-eGFP |
| 7. | pSP429 | Abi S166, 168, 169A (S3A) and HSPC300-eGFP |
| 8. | pSP430 | Abi S166, 168, 169D (S3D) and HSPC300-eGFP |
| 9. | pSP487 | ScarWT and AbiWT |
| 10. | pSP488 | ScarS8A and AbiS3A |
| 11. | pSP489 | ScarS8D and AbiS3D |

## References

1. Pollitt, A.Y.; Insall, R.H. Abi mutants in Dictyostelium reveal specific roles for the SCAR/WAVE complex in cytokinesis. *Curr Biol* **2008**, *18*, 203-210, doi:10.1016/j.cub.2008.01.026.
2. Ura, S.; Pollitt, A.Y.; Veltman, D.M.; Morrice, N.A.; Machesky, L.M.; Insall, R.H. Pseudopod growth and evolution during cell movement is controlled through SCAR/WAVE dephosphorylation. *Curr Biol* **2012**, *22*, 553-561, doi:10.1016/j.cub.2012.02.020.
3. Schaks, M.; Singh, S.P.; Kage, F.; Thomason, P.; Klünemann, T.; Steffen, A.; Blankenfeldt, W.; Stradal, T.E.; Insall, R.H.; Rottner, K. Distinct Interaction Sites of Rac GTPase with WAVE Regulatory Complex Have Non-redundant Functions in Vivo. *Current Biology* **2018**, *28*, 3674-3684. e3676.
